# Of rats and children: plague, malaria, and the early history of disease reservoirs (1898–1930)

**DOI:** 10.1007/s40656-024-00633-7

**Published:** 2024-10-22

**Authors:** Matheus Alves Duarte da Silva, Jordan Goodman

**Affiliations:** 1 University of St Andrews, St Andrews, UK; 2 University College London, London, UK

**Keywords:** Advisory Committee for Plague Investigation in India, Edmond and Etienne Sergent, John Stephens and Rickard Christophers, Healthy carrier, Disease ecology

## Abstract

This article’s jumping-off point is the highly incisive but often-ignored claim by the French doctor, Louis-Jacques Tanon, in 1922 that rats acted as plague reservoirs in Paris; in other words, that they harboured the plague bacillus but were refractory to it. This claim partially reframed the fight against this disease in the French capital in the 1920s, which became more centred on surveilling the plague reservoir rather than on destroying rats. Drawing upon Tanon’s hypothesis, this article explores the emergence, evolution, and several iterations of the idea of disease reservoirs in the early twentieth century. On the one hand, it describes the crafting of a range of ideas with which Tanon was directly or indirectly dialoguing, namely, that rats could present a stage called chronic plague, which was especially developed in India; and that human populations, especially children, acted as sources or reservoirs of malaria in Sierra Leone and Algeria. On the other hand, this article shows how Tanon created original reasoning by combining and reformulating some of these ideas and applying them to Paris. Thus, this article contributes to the early history of reasoning in terms of disease reservoirs, as well as presenting a more dynamic history of microbiology by showing how concepts crafted in the “Rest” found their place in Europe.

## Introduction

In September 1922, French medical doctors flocked to Marseille to attend the National-Colonial Congress for Public Health and Social Welfare [*Congrès colonial national de la santé publique et de la prévoyance sociale*].^1^ Among the speakers was Louis-Jacques Tanon. Assistant-professor [*chargé de cours*] at the Paris Institute of Colonial Medicine and medical-inspector of epidemics [*médecin-inspecteur des épidémies*] of the Paris Préfecture de Police, Tanon presented a paper on plague (Tanon, 1922). Caused by a bacillus known today as *Yersinia pestis*, plague provoked important outbreaks around the world from 1894 to 1950, which became described as the third plague pandemic (Echenberg, 2007). In the 1920s plague was still a global sanitary threat, affecting, for example, various French colonies—as other speakers insisted on (Heckenroth, 1922; Lalande, 1922; Le Dantec, 1922). In his talk Tanon argued that apparent healthy rats could be infected with a less virulent form of the plague bacillus and, therefore, constituted for plague what he called a “virus reservoir” [*réservoir de virus*] (Tanon, 1922, p. 250).^2^ This was not an unusual term for Tanon’s audience. French colonial doctors had been studying the problem of “virus reservoirs” since the early 1900s, namely malarial reservoirs in Algeria (Sergent & Sergent, 1905). In short, Tanon’s presentation was deeply tied to French colonialism and colonial medicine in almost every aspect—venue, audience, subject—but one. Tanon stressed that rats formed a plague reservoir not in any French colony, but in the centre of the empire: Paris (Tanon, 1922, pp. 249–251).

Tanon’s claim was likely one of the first times the concept of “virus reservoir” was applied to a contingent group of animals—Paris’s rats, in this case—in a European city. It is therefore an important, but overly ignored, landmark in the history of the concept of disease reservoirs, central in today’s epidemiological reasoning.

Reflecting upon contemporary uses of reservoir in medicine, Haydon and collaborators noted that “many different and often contradictory definitions of reservoirs exist” (Haydon et al., 2002, p. 1468). Ashford (1997, p. 86) had defined a reservoir of infection as “an ecological system in which the infectious agent survives indefinitely. Where a vertebrate host or group of hosts is essential to such a system, these are termed the reservoir host(s).” In response to Ashford, Haydon and collaborators proposed a more relative definition of reservoir, in which a target population should be defined beforehand. Thus, they defined reservoir “as one or more epidemiologically connected populations or environments in which the pathogen can be permanently maintained and from which infection is transmitted to the defined target population” (Haydon et al., 2002, p. 1469). More recently, reservoir hosts have been defined as “populations, species or ecological communities that drive disease dynamics because they can become infected by a disease agent and sustain transmission long enough to serve as a source of infection for uninfected hosts” (Salkeld et al., 2023, p. 133).

In most of these definitions, the authors highlight that the animals which constitute the reservoir are not necessarily refractory to the pathogen that infect them. However, these authors acknowledge that it could sometimes be difficult to demonstrate any harm caused by the pathogen to the reservoir (Ashford, 1997, pp. 89–90). This apparent resistance of the reservoir vis-à-vis the pathogen was central in Tanon’s reasoning and is probably today’s most popular interpretation of the concept. Indeed, as the Covid-19 and former pandemics of respiratory diseases have shown, vertebrate animals, like bats and birds, can potentially harbour known or unknown pathogens which do not necessarily cause them any harm (Hollingham, 2022; Jacobs, 2021). Given the real or potential sanitary risks, the management of animal reservoirs of disease has, in the past decades, called for an array of measures, including the separation of the reservoir from the target population, the surveillance of the reservoir, or even its destruction (Haydon et al., 2002, p. 1468; Keck, 2020).

Although pivotal in current epidemic and pandemic management, the origins and evolution of the concept of disease reservoir in the early twentieth century has not attracted much scholarly work until recently. One remarkable exception is Mark Honigsbaum’s study on California-based doctor Karl Meyer, who claimed in 1931 that the animal kingdom acted as a reservoir of diseases (Honigsbaum, 2016). As Honigsbaum puts it (2016, p. 266), “Meyer deserves to be seen as an important bridge figure in mid-twentieth century medical research that sought to link microbial behavior to broader bio-ecological, environmental and social factors that impact host–pathogen interactions and the mechanisms of disease control”. Honingsbaum (2016) locates the development of Meyer’s ecological approach in his exchanges with the English ecologist Charles Elton, American pathologist Theobald Smith, and the Australian immunologist Frank Macfarlane Burnet. More recently, historians have started to show other possible genealogies of reflections in terms of disease reservoirs, going beyond actors and institutions in the UK, USA, and Australia (Greatrex, 2023; Méthot, 2019; Silva, 2023). Also, some authors have shown that the concept of disease reservoir was not only applied to animals, but could refer to human populations, such as female workers and gay men as reservoirs of “venereal diseases” (McKay, 2023).

The work of Tanon on rats as plague reservoirs in Paris exemplifies the necessity for a more global picture of the first decades of the crafting of the concept of disease reservoirs. In reconstructing the intellectual world of Tanon, we aim to develop two parallel arguments. First, that Tanon engaged with ideas emerging in that period that aimed to explain a similar set of phenomena: the existence, and in some cases the persistence, of pathogenic organisms living inside the bodies of some animals, but also of some humans, without causing apparent harm to their hosts. “Healthy carrier” was one of these concepts, a condition articulated by Robert Koch at end of the nineteenth century during the Hamburg 1892 cholera outbreak and later applied by Koch to describe the epidemiology of East Coast Fever in Africa and typhoid in Germany (Gradmann, 2010). This discovery pointed out that healthy individuals could be spreading pathogenic micro-organisms, pushing for constant surveying and testing of healthy populations, such as cattle, in the case of East Coast Fever, or humans, when it came to typhoid (Mendelsohn, 1995). As we will see below, Tanon knew and referred to the concept of healthy carrier. Nonetheless, in his studies on plague his thinking related more closely to two other ideas: chronic plague, which he discarded, though, for the situation he had observed in Paris, and that of reservoir, which he embraced.

Both concepts of reservoir and chronic plague had been crafted in the early 1900s. The former appeared namely in Algeria, where it was used by the brothers Edmond and Etienne Sergent to talk about children harbouring the malarial pathogen, whereas that of chronic plague had been mobilised mainly by British colonial doctors to frame rats infected with plague in India. Therefore, our second argument is that in claiming that rats were a plague reservoir in Paris, Tanon was bringing and adapting to France ideas originally constructed by European doctors in some colonies in Africa and Asia. This shows an interesting pattern of transfers and circulation of microbiological knowledge, seldom discussed by historians. Simplistic diffusionist narratives in the history of science are no longer accepted (for an example of the diffusionist model, see Basalla, 1967). Nonetheless, we still have more histories on how microbiological ideas, practices, institutions and objects were first constructed in Europe and then exported, adapted, or imposed to the “Rest” (Latour, 1984; for a critical examination on the expansion of microbiology, see Velmet, 2020), than on how knowledge constructed in the “Rest” was adapted to Europe (as an exception see Gradmann, 2010, see also (Anderson, 2006) for the USA empire). Although the centrality of Europe in the emergence of microbiology is beyond doubt, this should not obscure the fact that at times knowledge constructed outside of Europe, even if by Europeans, made its way to the Old World, which, we argue, was the case with Tanon’s studies on rats as plague reservoir in Paris. In arguing this, we are not subscribing to a-critical or embellished visions of colonialism and globalization, as cautioned by Sarah Hodges and Warwick Anderson in their critical examinations of the global turn in the history of medicine (Anderson, 2014; Hodges, 2012). Instead, we aim to highlight the colonial origins of the disease reservoir framework and how this conceptualization took root not only in colonies, but in imperial centres.^3^

Alongside these two general arguments, in this article we aim to expand some aspects of the intellectual history of plague and malaria in the beginning of the twentieth century. The historiography of both diseases has in the last years addressed the problem of how some animals—rats and fleas in the case of plague, and *Anopheles* mosquitoes when it comes to malaria—were framed as vectors and spreaders of these two scourges (Deb Roy, 2013, 2017; Evans, 2018; Lynteris, 2022b; Webb, 2009; Winegard, 2019). Less attention has been paid to the “reservoirs” of these diseases (as exceptions, see (Lynteris, 2019; Silva, 2023). As we show, the research on the spread of malaria and plague rapidly transitioned to that of their pathogens existing undetected among apparent healthy humans and animals. This coalesced at the beginning of the twentieth century around the concepts that will occupy our attention here: malarial “reservoir”, “chronic plague”, and finally “plague reservoir”.

This article is divided into two main parts: one focussed on malaria, the other on plague. We start this article with the study of malaria by British doctors in Sierra Leone at the end of the nineteenth century, which pointed out that colonized children formed the “source” behind malaria endemicity. Then, we show how this insight was transformed into a “reservoir” of malaria in Algeria in 1905, in one of the first uses of this medical concept. In the second part, we shift from malaria to plague, from humans to animals harbouring diseases, and progressively from the South to the North. We describe the work carried out in India, from 1905 to 1910, on the possible existence of a so-called chronic plague among rats, later abandoned. Finally, we retrace Tanon’s research in the early 1920s, and how he braided together these earlier discussions unfolding in Sierra Leone, Algeria, and India in his claim of rats as plague reservoirs.

## Source of contagium

On the 13th of July 1898, Lord Joseph Lister, the President of the Royal Society of London, convened the first meeting of the Society’s Malaria Committee, a direct response to the request from Joseph Chamberlain, Liberal MP and Colonial Secretary, to form such a body to advise the Colonial Office on research into malaria in Africa (Chamberlain, 1898; Cranefield, 1991). By the end of the month, several key decisions had been made, particularly that two investigators, John Stephens and Rickard Christophers, would be spending some time in Italy to see for themselves the work that had been progressing there on malaria, and then to East Africa, to the hospital in Blantyre (now in Malawi), before continuing their investigations in West Africa (Royal Society Archives, 1898d, 1898e; Capanna, 2017; Snowden, 2006). The committee also decided that two representatives from the Colonial Office, Charles Lucas and Patrick Manson, would be invited to sit in on the committee meetings (Royal Society Archives, 1898a, 1898b; Bynum & Overy, 1998, p. 362; Worboys, 1988; Christophers, 1946; Shortt & Garnham, 1979).^4^ On 2nd August in a letter to the Colonial Office, the Royal Society spelled out their procedures. The letter reflected the views the committee members, almost all of them medical men, held about malaria. They agreed, on the basis of work conducted principally in Italy and Algeria, that malaria was caused by the presence in human blood of what they called “a minute parasite affecting the red corpuscles but that the organism’s life–cycle, especially the period it spent outside the body and its re-entry into it, was not fully worked out”. Critically, at this time, they were also of the opinion that the “view that the mosquito is an important agent in propagating the disease has at present advanced very little beyond the stage of hypothesis”. Therefore, they did not agree with Manson that an entomologist should be represented among the observers—determining the species was, in the committee’s opinion, not so difficult as to warrant the presence of “a special entomologist”. Perhaps it is not surprising that since most of the committee members were pathologists, they suspected that even though little was known about malaria in tropical Africa, “the important problems are pathological in nature” (Royal Society Archives, 1898c).

Stephens and Christophers sent their first report from Blantyre dated 15 May 1899—they had been there for over three months—and it did not make for positive reading (Royal Society Archives, 1899a). Basically, they felt that they were in the wrong place to study malaria for the simple reason that there were far too few cases with which to work (Royal Society Archives, 1899b). At the end of July 1899, Michael Foster, the Secretary of the Royal Society and of the malaria committee, wrote to Stephens and Christophers saying that “it is unfortunate perhaps for you that the whole malaria question has advanced so rapidly since you left England. At that time the ‘mosquito theory’ was still sub judice. Now the researches of [Ronald] Ross [in India] and the Italian observers have established beyond doubt that certain species do act as agents of infection”.^5^ Considering this new knowledge, Foster agreed that they had no reason to stay in Blantyre. They should return to England before embarking on their West African mission, as their original instructions intended for them.

Before they went to Freetown, their destination in West Africa, Stephens and Christophers spent some time in Liverpool where Ronald Ross had recently been appointed as a Lecturer to the newly founded Liverpool School of Tropical Medicine (Nye & Gibson, 1997; Power, 1999). By 5 February 1900, after a short visit with Ross, they were in Freetown (Royal Society Archives, 1900a). What they found on their arrival was a port–town, a British Crown Colony since 1808, of about 30,000 population, living partly on level ground extending from the sea and partly on the lower slopes of the surrounding hills. The rainy season lasted from May to November during which time malaria cases soared. The ‘White Man’s Grave’, an expression applied to the area from 1822, gave a clear sense of how unhealthy it was perceived by white settlers (Frenkel & Western, 1988, p. 214; Curtin, 1961, 1990). Europeans who lived in the central portion of the town represented less than 2% of Freetown’s population but it was their welfare that preyed most on Chamberlain’s and the Royal Society’s mind (Goerg, 1998, p. 3; Bockarie, Gbakima & Bashima, 1999, p. 213; Anon., 1898, p. 826).

In their report, published several months later, Stephens and Christophers contrasted conditions in Freetown between “well–built, clean houses” and “small dirty and dark houses and thatched sheds in which native boys (as many as twenty) slept” (Stephens & Christophers, 1900a, p. 46). They continued with their main point: “These overcrowded sheds were especially the abode of anopheles […] female [ones] in large numbers’ but in general ‘in native dwellings […] anopheles are frequently present in enormous numbers” (Stephens & Christophers, 1900a, p. 47). Indeed, as they remarked even more directly, casting their verdict over the local domestic arrangements: “we may look upon such a house and its accessory hovels as one infected with malaria, or as a ‘fever house”’ (Stephens & Christophers, 1900a, p. 57). Further along in their report, after discussing various potential methods by which the mosquito larvae might be killed, Stephens and Christophers returned to the subject of the “native”, no longer the abode but rather the person. They declared that “natives” were not immune to mosquito bites as others had stated, but, quite the opposite, they “attract mosquitoes, and especially anopheles, more readily than Europeans do. […] They powerfully attract anopheles” (Stephens & Christophers, 1900a, p. 56). As Clapperton Mavhunga (2011) has remarked, it was very common at the end of the nineteenth century and for a long time after that for British colonists to view “natives” as naturally constructed public health dangers and pests.

Having identified the “native” as a disproportional attractant to mosquitoes, Stephens and Christophers took this one step further. In early June 1900, the malaria committee received their next report. The title gave a powerful indication of how they were thinking about malaria as a socially transmitted disease: “The Native as the Prime Agent in the Malarial Infection of Europeans” (Christophers & Stephens, 1900). Now Stephens and Christophers narrowed their sights by identifying who specifically was responsible for initiating and sustaining the transmission of disease and, according to the two investigators, the culprit was children—“the source from which the infected anopheles, found in every house, derived their infection” (Christophers & Stephens, 1900, p. 12). Two other characteristics of the infection among children was revealed: infected children showed no or very little sign of disease—they ran about and were otherwise perfectly healthy; and as they got older, they showed increasing “immunity” to infection—cases of malaria in children over 12 years in age were rare (Christophers & Stephens, 1900, p. 15). These observations, Stephens and Christophers remarked, were very similar in structure to those the microbiologist David Bruce had argued was the way the animal disease called nagana (now referred to as trypanosomiasis) was transmitted in Zululand (Christophers & Stephens, 1900, p. 16).^6^ Just like malaria, whose vector was a mosquito, nagana was transmitted by a flying insect, the tsetse fly, which became infected with a parasite by biting wild game and subsequently infecting domestic animals by biting them. Put another way, children, in the case of malaria, were the equivalent of wild game in the case of nagana. In establishing this link between nagana and malaria, the research of Stephens and Christophers suggests that discussions and intellectual exchanges between colonial doctors working in different parts of the British Empire and on different topics was fairly normal.

Stephens and Christophers continued on the theme of malaria and children in the first of two further reports, which contained highly technical details, that the committee received on 1 October 1900. Basing their further observations on the microscopical examination of blood samples for the presence of parasites, they continued to attribute the transmission of malaria to children who, they wrote, “are constantly exposed to infection from anopheles” (Stephens & Christophers, 1900b, p. 9). In the second report, the two investigators proposed what they thought was the most efficacious method of reducing the incidence of malaria in the resident European population. They began their report by referring to the famous microbiologist Robert Koch’s observations in German New Guinea of the substantial incidence of malaria among children (Anon., 1900; Verhave, 2022).^7^ Koch pointed out that the incidence of malaria parasites in blood smears, as a percentage of the total smears examined taken from children under the age of ten, acted as a marker of the degree to which malaria was endemic in a particular locality. In their research, Stephens and Christophers came to the same conclusion. They called Koch’s marker an “index of the endemicity of malaria” (Stephens & Christophers, 1900c, p. 21).^8^ As long as there were “native” children present who showed no symptoms of malaria infection, the prevailing condition, on the contrary, was “one of a constant infection (our emphasis) […] a state of things parallel to that of trypanosoma in wild game and rats” (Stephens & Christophers, 1900c, p. 22). Europeans, they argued, do not “derive malaria from pre–existing cases in Europeans” (Stephens & Christophers, 1900c, p. 22). Europeans, they went on to say, “must recognize that malarial fever is a contagious disease [contracted] through the medium of the mosquito from the native child. […] [It] can be avoided most readily by avoiding this source of contagion and living as far removed as possible from native huts” (our emphasis) (Stephens & Christophers, 1900c, p. 23). “The question, they concluded, is not so much how large towns with sometime 40,000 native inhabitants can be freed from anopheles, but how the relatively small number (at most 200) of Europeans can be protected” (Stephens & Christophers, 1900c, p. 23). “In Africa, they wrote, a complete isolation of Europeans would, we believe, render malaria a comparatively rare disease” (Stephens & Christophers, 1900c, p. 24). In Freetown, in effect, this meant building an entirely new residential neighbourhood exclusively for Europeans in the surrounding hills without any nearby “native quarters” (Stephens & Christophers, 1900c, p. 24).^9^ Segregation was then the only solution.

By the time the Royal Society malaria committee received these reports from Stephens and Christophers, they had already been converted from scepticism to certainty when it came to the mosquito-malaria theory. In a letter to the Colonial Office dated 24 February 1900 they expressed this new-found understanding. One passage stands out as an insight into the dynamic entanglements that recent research had revealed between the parasite, the mosquito, and the human. It is worth quoting in full:

A person suffering from malaria is, so long as the parasite is to be found in the blood circulating through the skin, an infectious person. If bitten at this time by an anopheles, he will communicate the disease to the insect, and so propagate the infection. In this way a single person going to a district where the anopheles were previously free from parasites, may start the disease among the insects and so prove the origin of an epidemic among men (Royal Society Archives, 1900b).

To sum up, the assumption that children played an important role in the epidemiology of malaria in Sierra Leone, because they literally “infected” mosquitoes, which in their turn transmitted the disease to Europeans, opened new avenues for colonial intervention including town planning and segregation. Moreover, it also created momentum for original investigations and pushed for explaining the medical state of these colonized children vis-à-vis malaria. In other words: how to conceptualize these children who seemed almost immune to malaria, which Stephens and Christophers referred to as source of contagium?

## The emergence of reservoirs

Stephens and Christophers pointed the finger at the “native child” as being responsible for how this “contagious disease” was contracted. They advocated that Europeans should segregate themselves if they ever hoped to avoid being infected. In Algeria, too, children were being viewed by those studying malaria as the source of the disease. The main French colony in the Maghreb had already a long tradition on malaria studies. It was there that Alphonse Laveran discovered in 1880 the existence of a living microorganism in the blood of a malarial patient in the military hospital in Constantine, and named plasmodium (Smith, 1985). The discovery in this place was not a coincidence. The surrounding area was highly malarial and that condition prevailed throughout the Algerian lowlands stretching inland from the Mediterranean coast in a westerly direction towards the present border with Morocco.^10^ The area’s unhealthiness was, according to Jean Baptiste Paulin Trolard, a leading French anatomist working in Algiers and co–founder of the Pasteur Institute in Algeria, “the largest obstacle to colonization” (Trolard apud Fredj, 2016, p. 294).^11^

In 1903, Edmond Sergent, at the time a colonial doctor, agreed completely with this statement—indeed, he virtually repeated it—but he also added that malaria also severely limited the ability of Algeria to develop (Sergent, 1903, p. 35). Sergent was born in Philippeville (now Skikda), Algeria, to French parents (Dedet, 2013; Strachan, 2006). He trained in medicine in Algiers and then continued his studies in microbiology and entomology in Paris (Dedet, 2008). His brother Etienne, who was almost two and a half years younger, followed his brother’s educational pattern (Dedet, 2013). Of the two brothers, it was Etienne who published first, with an article that appeared in 1901 on the presence of Anopheles on the banks of the Essonne river, a tributary of the Seine, to the south of Paris, during the summer months, where no cases of malaria had been known for some time (Sergent, 1901; Sergent & Sergent, 1903).^12^ Edmond wasted little time keeping up with his younger brother with a publication in the following year on the presence of a new parasite in the blood of a chameleon (Sergent, 1902). It is not surprising therefore that in addition to their own publications, the two brothers joined forces and began publishing together, first on mosquitoes in the Paris suburbs and then about malaria in Algeria (Sergent & Sergent, 1902).

In the summer of 1902, Edmond and Etienne, at the instigation of the governor–general of Algeria and with the full support of the Pasteur Institute in Paris, were in Algeria to initiate an antimalaria campaign beginning with a test–case, to see if there was a satisfactory method by which Europeans could be defended against Anopheles (Sergent, 1903, p. 39). The only guiding principle for deciding on a suitable test site was that it had to be extremely malarial; and that turned out to be the station of Alma, 40 kms east of Algiers on the rail line from the latter to Constantine (Moulin, 1996, pp. 170–171). As Edmond remarked, every one of the past station masters, including the present one, had suffered from malaria, their incapacities leading, in all cases, to time off work and even to re-assignment (Sergent, 1903, p. 39). Edmond and Etienne decided that they would try out the current techniques that were being used elsewhere, primarily in Italy and in the United States in what they called “a war against mosquitoes”—especially protecting houses and individuals from mosquitoes and their bites, using netting wherever possible; and applying petroleum products, such as kerosene, to the surfaces of stagnant bodies of water to kill mosquito larvae (Sergent, 1903).^13^ The result of this small experiment was a complete success and they felt confident that the techniques could be used over a wider area (Sergent, 1903, p. 63–65). A year later, and on the basis of the trial run, the director of the rail company serving the station of Alma asked the Sergent brothers to repeat their preventative measures at seven other stations on his line. The incidence of malaria fell there as it had done in Alma the year before (Sergent & Sergent, 1928, pp. 123–124).

In 1904, Edmond and Etienne Sergent were effectively put in charge of the campaign to eradicate malaria from Algeria (Dedet, 2008; Fredj, 2016). In that year, the brothers set about examining an area that was new to them, but which was known to be particularly malarial, that is the region that spread inland from the coast to the west of Algiers, the Mitidja Plain. They spent most of the year 1904 travelling, observing, and testing for the presence of malaria. Although they did not cite any authors in the report in which they described their observations and which they published in the following year, it is clear that they were influenced by Koch’s observations on malaria made during his trip to German New Guinea in 1900 in which he pointed to the importance of the percentage of children infected with malarial parasites as a gauge of how endemic the disease was in a specific place.^14^ Over the year and across a number of settlements on the plain, the brothers calculated what they called an “endemic index” [index endémique], the proportion of local, non–European children (not older than fourteen), in whose blood they observed the presence of the malaria parasite. That figure, which effectively measured the degree of the presence of malaria, varied throughout the year but in the summer months reached as high as 85% (Sergent & Sergent, 1905, p. 130).

So far, there was nothing novel in their approach to studying malaria. The “endemic index” was useful in isolating pockets of disease but little more than that. But the Sergent brothers now proposed that what they called the “virus reservoir” [*réservoir de virus*] which provided a conceptual tool for embarking on an antimalarial programme.^15^ In their reasoning, the reservoir was a pool of humans. It consisted of two groups: Europeans who had been infected with a symptomatic version of malaria and in which the plasmodium continued circulating in their blood, and in the minority; and local children, “often infected without morbid manifestations”, by far the majority and who represented the bulk of malaria infections in a community (Sergent & Sergent, 1905, p. 129). The incidence of malaria in any community will be reduced, they importantly asserted, by reducing the size of the reservoir alone, that is without taking any further action (Sergent & Sergent, 1905, p. 133). To make their case, they offered evidence from several places, including a train station and several villages where malaria and indigenous people were present: when the latter were either removed or separated from Europeans, the cases of malaria fell dramatically (Sergent & Sergent, 1905, pp. 134–136).

To the brothers, the reservoir acted as a constant and dangerous source of infection. Any antimalarial campaign had to act on this reservoir and reduce its potential of infectivity, by removing it or moving away from it; and by cleansing it of parasites by administering quinine to the population constituting the reservoir.^16^ Without these measures, the reservoir, which could persist for a long time if not treated, would continue to infect the mosquitoes, the transmitters of the malaria parasite and ensure the existence of the epidemic. In short, as Stephens and Christophers had argued just a few years before in the case of Sierra Leone, the Sergent brothers were advocating, by mobilizing the idea of reservoir, for segregation in Algeria in the name of public health concerns.

In the first decades of the twentieth century, the idea of a disease reservoir became progressively central to managing malaria, being applied in Algeria to human populations, namely colonized children, rather than programs designed to eradicate mosquitoes, an approach advocated most forcefully by Ronald Ross and his supporters.^17^ In the next sections, we chart how this concept jumped from malaria to plague and was mobilized to justify a continuous “surveillance” of rats in Paris. Before examining the application of the idea of reservoir in Paris, we will head back to the British empire, discussing ideas crafted in India—that of chronic and resolving plague—that also informed the later research on plague reservoirs in France.

## From chronic to resolving plague

In 1894, plague reappeared within the European colonial world, in Hong Kong, and soon spread over all inhabited continents (Echenberg, 2007). Retrospectively described as the third plague pandemic, it caused havoc in various parts of the globe, killing more than 12 million people by the 1950s, most of them in India (Arnold, 1993, Chap. 5). The pandemic perpetrated an all-out war on rats, which was achieved, namely, through direct destructive actions—such as hunting, poisoning, trapping, and fumigation—and by rat-proofing infra-structures (Vann, 2003; Silva, 2016; Engelmann & Lynteris, 2019; Jules Skotnes-Brown, 2023; Silva, 2024). Therefore, it is not an exaggeration to state that rats, along with mosquitoes, became the most destroyed animal in the world in the first half of the twentieth century.

Until the Hong Kong outbreak, rats were seldom associated with plague, and their destruction was seldom justified on sanitary grounds (Lynteris, 2022a; Pemberton, 2014). The connection between these animals and plague began to be bridged when it was observed, first in Hong Kong and then in further outbreaks, that rats usually died prior to human cases of plague and that in the bodies of a few specimens the plague bacillus could be found (Kitasato, 1894; Yersin, 1894). Nonetheless, their exact role in spreading plague remained a matter of debate at the end of the nineteenth century. Doctors such as Alexandre Yersin, who described the plague bacillus for the first time, wondered if rats were being infected through the soil, which made them a kind of first victims, or sentinels, but not necessarily responsible for the human cases (Lynteris, 2017). When plague reached India in 1896 new hypotheses emerged, namely that rats infected humans via their dejections or that rat fleas transmitted the bacillus among rats, and from rats to humans (Hankin, 1898; Simond, 1898). The latter idea is commonly attributed to the French doctor Paul-Louis Simond, who, from June 1897 to August 1898, was in a mission at the Presidency of Bombay to test anti-plague sera developed at the Pasteur Institute in Paris. Nonetheless, the hypothesis of the rat flea as the plague vector was fiercely criticized both in India and in Europe, because Simond’s experiments were few in number, he had been unable to identify the species of fleas he encountered, and for the epistemic problems of transferring conclusions from rats to humans (Silva, 2020, pp. 269–274).^18^

In 1904, amidst this controversy on the spread of plague and with cases and deaths skyrocketing, the Government of India decided to organize a commission, dubbed the Advisory Committee for Plague Investigation in India (hereafter ACPII) (Martin, 1905).^19^ This commission had many points in common with that of Stephens and Christophers on malaria. Indeed, to put its plan into action, the Government of India sought the expertise of the Royal Society, “because it had experience in sending out and managing similar scientific expedients for scientific research”; and that of the Lister Institute, the main bacteriological institution in Britain (Royal Society Archives, 1904b). Combining fellows from both institutions and doctors based in India, the ACPII aimed to answer a very precise question: Did the plague bacillus enter the human body via “chance abrasions” or was it “inoculated by the bite of an insect”? (Royal Society Archives, 1904a). The experiments began in India in 1905, split between Bombay and the Punjab, and occurring in laboratories, villages, and cities, and involving the capture and examination of hundreds of rats and fleas (Evans, 2018). After one year of intense work, the ACPII concluded that fleas spread the plague bacillus among rats (Lamb, 1906, p. 2). Furthermore, the ACPII established that the rat flea could bite humans and live from their blood (Advisory Committee, 1907b). These observations led ACPII’s president Charles Martin to conclude “that the rat flea and the rat flea alone is the agent of transmission of plague from rat to man in infected houses”, even though an experiment in that sense was not carried out, since this would almost certainly be a condemnation to death (Martin, 1908, p. 2). ACPII conclusions, published in the form of reports and as articles in the *Journal of Hygiene*, reached great success in India and abroad, becoming the gold standard of plague epidemiology in the following decades and justifying in many cases the war against rats (Audoin-Rouzeau, 2003; Evans, 2018).

ACPII also carried out studies, among other subjects, on what happened with the plague bacillus between epizootics and epidemics. The investigations began in 1906, with the observation that among hundreds, and even thousands of healthy rats captured in endemic hotspots, it was possible to find lesions and abscesses in a very small percentage of them. In general, these animals did not present any apparent symptoms of plague, but further bacteriological analysis showed that the lesions contained plague bacillus, a condition the ACPII called chronic plague (Advisory Committee, 1906, 1907a). The ACPII argued that chronic plague could explain the seasonality of the disease in parts of India. As the ACPII noted, whereas plague affected rats all year round in Bombay, in the Punjab, on the other hand, it seemed to disappear from these animals until the next epizootic. Therefore, the ACPII wondered whether, in the Punjab, the plague bacillus continued to survive inside rats during the interval of the epizootics in this chronic form, and if a causal relationship existed between chronic and acute phases. The ACPII advanced a few hypotheses on the possible connections between them. For example, the commission noted that rats consuming the carcasses of other rats affected by the chronic plague would be infected by the microbes contained in the abscesses, and therefore an epizootic might follow. However, these discussions remained at a hypothetical level, the ACPII not providing any definite conclusion (Advisory Committee, 1907a).

In 1910 the ACPII abandoned its earlier conclusions about chronic plague in favour of a different hypothesis. After thousands more dissected rats, it wrote that “the pathological appearances we have described as chronic plague are the stages in the process of recovery from the acute disease” (Advisory Committee, 1910, p. 335). Therefore, the ACPII suggested replacing the term chronic plague with what they called “resolving plague”. Far from being only a semantic change, the idea of resolving plague had implications in the broader plague epidemiology framework, by not ascribing a significant latent moment for plague among rats, or to put it differently, to not imagining rats as reservoirs of plague in the sense attributed by the Sergent brothers to children in relation to malaria. In the framework crafted by the ACPII, a new epizootic leading to a plague outbreak would mainly be caused by either the arrival of infected rats to a locality free from plague, as it seemed to be the case in the Punjab, or by the birth of new rats in cities where plague never ceased to exist among rats, such as in Bombay (Advisory Committee, 1912).

The works of the ACPII were translated and commented on in France by the *Office International d’Hygiène Publique* [OIHP], the first health organization with a global remit, created in 1907 (Anon, 1909). OIHP’s secretary Henri Pottevin affirmed that “the experimental and epidemiological research developed in the last years […] showed the preponderant role on the propagation of plague played by: 1) rats, as ambulant reservoirs of virus [*réservoirs ambulants de virus*]; and 2) fleas, as agents of dissemination of the infectious micro-organism, which they catch from the moribund rat and infect, according to the circumstance, a rat or a man [sic]” (Pottevin, 1910, p. 542). In this formulation, reservoir referred to the fact that the rat body contained a given microorganism that could kill it or at least provoke serious lesions, being in this sense, distant from the Sergent brothers’ reasoning. In 1920, Bordeaux-based doctor Raymond Sigalas published a thesis on the rat as a reservoir of a plethora of human diseases. To Sigalas, the term ‘reservoir’ harboured a range of meanings, spanning from the rat as source of human diseases to which it was immune (leptospirosis, for example) to the rat as a spreader of human diseases that also killed it (plague, for example) (Sigalas, 1920, pp. 17–18 and 44). Therefore, by the end of the 1910s the idea that rats could act as reservoir of plague was already in discussion among French doctors, although the edges of this concept were blurred and its meanings almost contradictory, when compared with other definitions of reservoir. Tanon’s research on plague interacted with this stream of studies but with a twist.

## Reservoir rats

The French Empire was affected by plague since the first years of the pandemic (Heckenroth, 1922). In the metropolis, the fear that plague would make an appearance was rife by the end of the nineteenth century, which justified rat destruction and rat examination in the main French ports in 1898. In the following years, plague epizootics among rats and human cases were detected in French ports, mainly in Marseille, but no significant plague outbreak was declared in France until the end of WW1 (Pellissier, 1902; Sigalas, 1920, pp. 46–47).

This epidemiological situation changed in the first days of June 1920, when cases of plague were diagnosed among patients living in the ragpickers’ slums near Clignancourt’s gate, in the north of Paris (Bordas, 1923, p. 73). In the next weeks, the disease spread into parts of the 19th and 20th *arrondissements* of Paris, and to the adjacent cities of Clichy, St-Ouen and Levallois (*Note Confidentielle*, 1920, fol. 1) To fight the outbreak, the sanitary authorities of the Préfecture de Police relied mainly on rat destruction, therefore reproducing in Paris measures applied in several cities around the world, from Rio de Janeiro to Hanoi (Bordas, 1923, p. 74; Vann, 2003; Silva, 2024). The Parisian population was encouraged to kill and deliver rats to the health authorities, for which a reward of 0.25 francs per animal was paid. In parallel, the Préfecture de Police oversaw the deratization of structures containing large human populations, such as prisons and military barracks. Together, these measures allowed the destruction of more than one thousand rats per day during a period of almost 18 months (Bordas, 1923, pp. 79–80; Tanon, 1923, pp. 611–613). By the end of 1920, the disease had already provoked 92 cases and 34 deaths (Joltrain, 1921, p. 71). Although already under control by early 1921, the outbreak was officially declared over only in October 1921; in January 1922 the wide anti-rat campaign ended (Tanon, 1923, pp. 611–612).

In July–August 1920, the Préfecture de Police created a laboratory where rats were autopsied in search of the presence of plague bacillus. As described by Frédéric Bordas, general inspector of the technical services of hygiene in Paris, the microscopic and bacteriological examinations of rats were done in order to “forecast the diffusion of plague” in Paris. Knowing where plague was more widespread among these animals, the sanitary service could increase the anti-rat fight in those areas (Bordas, 1923, p. 77). The new institution was officially called Plague Prophylaxis Laboratory [*Laboratoire de Prophylaxie de la Peste*], but it was usually referred to as the Rat Laboratory [*Laboratoire des Rats*] (Bordas, 1923, p. 77; Tanon, 1923). Tanon directed the laboratory since its creation (Tanon, 1928, p. 5).

Louis-Jacques Tanon is largely overlooked in accounts of the history of medicine in France. Born in 1876 in a wealthy and well-placed family—his father was a member of the French Supreme Court—he became doctor in medicine at the Faculty of Paris in 1908 and assistant-professor in the same institution in 1913. During his training, Tanon worked as a laboratory assistant [*préparateur*] in the Vaccine Institute and taught hygiene and colonial diseases, with a particular focus on cholera and malaria, at the Institute of Colonial Medicine (Tanon, 1928, p. 5). In WWI, he had the opportunity of observing first-hand and treating warm climate diseases among black troops fighting in Europe. Once the hostilities finished, he resumed his teachings on colonial diseases in Paris and worked at the Claude Bernard Hospital, where the plague cases found in Paris were isolated. In 1922 he became medical-inspector of epidemics at the Préfecture de Police and two years later chief-inspector. In 1928 Tanon was hired as professor of hygiene at the Faculty of Medicine in Paris, occupying then leading academic and political positions, including the presidency of the French Academy of Medicine in 1964. Tanon died in 1969 at the age of 92 years (Anon, 1928; Tanon, 1928, pp. 3–9; Lecomte, 1935, p. 20; Huguet, 1991, p. 644).

On 12th September 1922 Tanon shared the conclusions of the works carried out at the Rat Laboratory at the Colonial Congress in Marseille, with which we opened this article (Tanon, 1922). Tanon began his talk by explaining that among the more than 5,000 rats he had examined microscopically and bacteriologically from July 1921 to January 1922, when the outbreak was already receding in Paris, in only 29 of them, that is approximately 0.5% of the total examined, had he found the plague bacillus (Tanon, 1922, pp. 250–251). But these 29 rats appeared healthy: they did not have any apparent symptoms of plague nor had their organs presented any lesions or scars, and only rarely was it possible to find small buboes in them. It was only by removing small parts of the rats’ liver and spleen, cultivating the microbes found in them, and then injecting these cultivations in laboratory animals that later died of plague, that it was possible to conclude that those rats were harbouring the plague bacillus (Tanon, 1922, p. 251–252). Therefore, Tanon advanced the hypothesis that, for plague,“the rat appears as a reservoir of virus” (Tanon, 1922, p. 250). This hypothesis could explain, according to Tanon, “better than the always dubious search for a new importation every time an outbreak occurs, how plague can reappear in a locality previously infected, one or two years after its disappearance. The enzootic outlives the epidemics, under an attenuated form that escapes the examination, and that can survive for a long time” (Tanon, 1922, p. 253).

Tanon thus wondered why these rats had become a plague reservoir. He firstly discussed the possibility of these animals having an “acquired immunity”, thanks to an infection in their youth or by a benign infection in a later stage of their development. But he discarded these hypotheses, arguing that immunity by “plague was definitive and durable” (Tanon, 1922, p. 253). Instead, he backed the idea that the plague microbes harboured by the rats he examined were less virulent and had not caused a previous benign infection. However, he did not or could not explain why the virulence of these microbes had been reduced. Nor could he explain the mechanisms by which the plague bacillus regained its virulence. As the ACPII did more than 10 years before in a similar subject, Tanon only advanced some possible explanations, conjecturing whether the passage through the flea increased the bacillus’s virulence, or whether new-born rats with no previous immunity could trigger an epizootic (Tanon, 1922, p. 254).

Tanon’s argument on rats as a plague reservoir can be viewed as being within the stream of research exploring the problem of pathogens surviving undetected among some human and animal populations, and not necessarily causing harm to their hosts, which, as we have seen, had coalesced around different concepts. To start with, Tanon mentioned the idea of “chronic carriers of bacillus” [*porteurs chroniques de bacilles*] when talking about the rats he found plague-infected in Paris (Tanon, 1922, p. 250). However, Tanon emphasized differences between “carriers” and “reservoirs”. He insisted that whereas the former carried “a microbe always dangerous to its neighbours” the rats he examined “presented an attenuated bacillus, weakly virulent”, and therefore, were not always dangerous to other rats (Tanon, 1922, p. 250). Tanon also discussed the idea of chronic plague, directly mentioning the ACPII. Nevertheless, he stated that the British commission in India had found lesions among the rats, whereas the rats he examined presented no lesions, scars, or abscesses, and only thanks to bacteriological examination was it possible to conclude that their organs contained the plague bacillus. This suggested that the plague bacillus had not caused any harm to these animals while infecting them. Therefore, it was not in the idea of carrier or chronic plague, but that of reservoir that Tanon found the best framework to describe the phenomenon observed in Paris (Tanon, 1922, pp. 250–251).

Tanon (1922, p. 254) defined a disease reservoir “as organisms which, although harbouring a pathogenic microbe, are refractory to its action in the usual conditions. The agent [the microbe] does not find in them a proper milieu [*millieu*, in the original] to its multiplication or its exaltation until that an external cause weakens or transforms the terrain [*terrain*, in the original]”. As we have seen, other French doctors, such as Pottevin and Sigalas, had previously claimed that rats acted as plague reservoirs but understood it differently to Tanon: for them, the rat was not refractory to the plague bacillus. Henceforth, the main insight brought by Tanon was to apply an understanding closer to that of the Sergent brothers, i.e., that a given population of rats could harbour the plague bacillus without being necessarily affected by it. Nonetheless, some differences between the Sergent brothers’ and Tanon’s reasonings should be stressed: for the formers, the malarial pathogen became unharmful to its reservoir after a first infection, which could be symptomatic and with lesions, as for European colonists, or almost unharmful, as in the case of colonized children in Algeria. But after this first encounter, the malarial pathogen did not cause any subsequent harm. To Tanon, rats could harbour plague without developing any external or internal symptoms. However, the plague bacillus could regain its virulence and kill its reservoir following external changes.^20^

Tanon’s original talk was published in the minutes of the Colonial Congress; and an extended version appeared in *La Presse Médicale*, co-signed with Bordas and Henri Dubief, both Tanon’s superiors at the Prefecture de Police’s hygiene services (Bordas et al., 1922; Tanon, 1922). In 1928, an enlarged version of the original paper was presented by Tanon at the Rat Conference (*Conférence du Rat)* held in Paris. This second version was published in the journal *Annales d’Hygiène Publique, Industrielle et Sociale*, co-signed with Bordas and Raymond Neveu, the new director of the Rat Laboratory following the promotion of Tanon (Bordas et al., 1928). In short, the rat as a plague reservoir was a hypothesis mainly backed by doctors attached to the Paris Préfecture de Police’s sanitary services.

Precisely because of this institutional link, the new conceptual framework of the rat as a reservoir of plague refashioned the fight against plague mainly in Paris (Bordas et al., 1931, p. 605). Starting in 1922, the Rat Laboratory began “surveilling” the reservoir, which was achieved by setting rat traps two or three times per year, and only in neighbourhoods where plague cases had been previously identified and in buildings where a rat infestation was communicated to the Préfecture de Police (Tanon, 1923, pp. 610 and 616). Every rat caught was dissected, examined microscopically and bacteriologically (Bordas et al., 1928, p. 379). The change of politics can be measured objectively: whereas in 1921 the Rat Laboratory examined 14,765 rats, between 1922 and 1930 this number plunged to around 2000 examined *per year,* which seems to indicate the reduction in the number of rats destroyed (Bordas et al., 1932, p. 344). But some of the previous politics remained in place in the 1920s: for example, if the presence of plague was confirmed in a rat or a human, the sanitary authorities pursued a number of preventative measures in the infected area, including vaccination, disinfection, and deratization (Bordas et al., 1928, p. 379; Tanon, 1923, pp. 608–611). Between 1922 and 1927, the Rat Laboratory found 120 rats infected with plague in Paris and its surroundings, and 27 cases of plague were confirmed among humans. However, no outbreak was officially declared in the French capital in this period (Bordas et al., 1928, p. 380). The absence of outbreaks was explained as a direct consequence of the precautions taken against the plague reservoir (Bordas et al., 1932, p. 344). Drawing upon his Paris experience in the 1920s, Tanon advocated (1929, p. 60) that plague prophylaxis should aim for “(1) the protection of healthy individuals; (2) the destruction of rats and fleas; and (3) in the places where plague has existed before, the surveillance of rats” (our emphasis).” Tanon was thus suggesting an adaptation to plague management: not only centred on rat destruction and on avoiding contact between rats and humans—which had been the main strategies adopted since the early 1900s—but also the constant surveillance of plague reservoir, by examining apparent healthy rats.

In France and in the French empire, Tanon’s argument that the rat was a reservoir of plague was well received. Already by 1922, his conclusion was quoted and endorsed by Georges Ribot, Marseille’s Director of Public Health, who believed it could explain the recurrent apparition of plague among rats in Marseille’s port without any “foreign” importation (Ribot, 1922).^21^ Doctors in Senegal and Morocco also agreed with Tanon and integrated his hypothesis into their own work on plague epidemiology (Leger, 1923, p. 312; Colombani, 1924, p. 576). In Tunis, the idea of the rat as a reservoir of plague was even mobilised to justify the functioning of a laboratory having the Paris Rat Laboratory as its model (Reynal & Wassilieff, 1932, p. 182). In other parts of the world, Tanon’s idea was not unknown, but its reception had been less enthusiastic (Uriarte & Villazon, 1928, p. 167). In an official summary of the most up-to-date knowledge on plague published in 1929, British expert George Ford Petrie claimed that Tanon’s idea had “lost contact with accepted knowledge, and [was] scarcely credible”. To deny it, Petrie pointed out that research carried out in South Africa had been unable to reproduce Tanon’s claim (Petrie, 1929, p. 193). Petrie’s criticism suggests the longstanding influence of ACPII’s conclusions that rats were never refractory to the plague bacillus. Furthermore, Tanon and collaborators complained that their work had been ignored in international forums and the hypothesis of the rat as a reservoir of plague never discussed at the assemblies of the OIHP and the Hygiene Committee of the League of Nations (Bordas et al., 1928, p. 377). This mixed reception of Tanon’s ideas suggests, perhaps, that not all microbiological knowledge crafted in Paris became global.

## Conclusion

This article has contributed to the small but bourgeoning historiographical literature on the emergence and evolution of the concept of disease reservoir, and to the broader debates on the history of ecological approaches to diseases. Regarding this last subject, an interesting historiographical debate has arisen in the last years regarding these questions: did microbiologists have ecological conceptions before the 1920s (Mendelsohn, 1998), or was disease ecology developed in the 1930s thanks to dialogues with modern ecology (Honigsbaum & Méthot, 2020)? Without entering into the merit of disease ecology *avant la lettre*, we have instead taken a different path, choosing to situate Tanon’s claim that rats acted as plague reservoir in its intellectual context. In so doing, we showed Tanon’s direct and indirect interventions in other concepts emerging in the first quarter of the twentieth century, including healthy carrier, chronic plague, and reservoir. These terms pointed to an analogous phenomenon: that pathogens were not only involved in killing their hosts, but they could also survive in a sort of balance among given populations to which they were or became unharmful. Thus, Tanon’s dialogue with these concepts seems to confirm, on the one hand, Anne-Marie Moulin’s (1991, p. 230) insight that the discovery of the carrier state “nuanced the omnipotence of the microbe and reinforced the importance of the terrain [*terrain,* in the original]”, i.e., of a medical reasoning closer to that of Claude Bernard. On the other hand, Tanon’s reasoning suggests the emergence of a more holistic and environmentally oriented view of microbiology before 1920. The concept of reservoir pointed out that the equations “microbe + host = death of the host” or “microbe + vector + host = death of the host” was much more complex and involved several other ecological elements.

This article has also demonstrated the multifarious, and even ambiguous and contradictory uses of the term “reservoir” in the first quarter of the twentieth century. Initially applied to frame children exposed to so-called benign forms of malaria in Algeria, the term underwent a threefold transformation in the 1910s and early 1920s: it became applied to animal populations, to animal populations in Europe, and to animal populations that might or might not be harmed or killed by a pathogen. This past confusion and contradictions may not surprise today’s researchers on disease reservoirs.

Finally, this article has insisted on the colonial background of the concept of disease reservoir. It was argued that the emergence of this and the other medical ideas discussed in this article were not only a purely intellectual adventure: these new intellectual frameworks were entangled with imperial expansionary projects and sanitary anxieties on how to protect British and French colonists from pathologies found in the colonies. The doctors involved in these different branches of research were either White settlers, such as the Sergent brothers, or deputations dispatched by the metropolis, as in the case of the malaria and plague commissions sent to Sierra Leone and India, respectively. In short, they were not examples of indigenous actors, in a very strict understanding of this concept, nor were these concepts examples of indigenous ontologies. However, these European doctors did not bring the concepts from Europe and simply apply them to Africa and India. Rather, these doctors crafted these concepts in the colonies. Later, other doctors adapted these concepts to Europe, as Tanon’s research indicated. To sum up, the discovery and management of the plague reservoir in Paris suggests that the colonial history of microbiology should be taken into consideration also when examining the development of the science of microbes in Europe.

## Footnotes

1 The congress occurred in parallel with the National-Colonial Exposition [*Exposition Coloniale Nationale*]*.* On both events, see (Régismanset, 1921; Congrès, 1922).

2 The term “virus” in 1920 did not have today’s meaning. At the turn of twentieth century, virus could refer to poison, to bacteria, or to filterable microorganisms, such as those causing rabies or smallpox – these two are today considered as virus. Tanon was applying the idea of “virus reservoir” to the plague bacillus, a bacterium. On the meanings of virus at the turn of twentieth century, see Duclaux, 1898, pp. 32–37.

3 The insight on the colonial roots of the disease reservoir framework was first presented in a collective-written introduction to a special issue on disease reservoirs, co-signed by one of us (Silva et al., 2023).

4 Manson had had a celebrated career in what was just being called “tropical medicine”. In 1897, Chamberlain had appointed him to be Medical Adviser to the Colonial Office (Chernin, 1992; Haynes, 2001). Stephens was born in 1865, and Christophers, in 1873.

5 Though not specifying which, Foster would certainly have had the following publications in mind: (Manson, 1898; Ross, 1898, 1899). As for Grassi and his colleagues in Rome, Foster would certainly have been thinking about (Grassi, 1898), which identified the mosquito species causing human malaria and which he had had translated for Stephens and Christophers; and a critical publication (Bignami, 1898a, 1898b). In 1896, Grassi had been awarded the Royal Society’s Darwin Medal.

6 Though they gave no references, Christophers and Stephens must have been referring to (Bruce, 1896), a continuation of earlier work on the same subject (Bruce, 1895). See also (Nuttall, 1899, pp. 68–71; Brown, 2008).

7 Details of Koch’s New Guinea trip, including his observations about children and malaria, appeared in the *Deutsche Medizinische Wochenschrift* on 26 April and 3 May 1900. Stephens and Christophers cited these in their report. An English translation appeared as (Anon, 1900).

8 To what extent the use of this quantitative measure was applied to other diseases is a discussion that is beyond the scope of this article. In his recent study, Jacob Steere-Williams discusses endemicity of typhoid as pertaining to soil very briefly at the same time as referring to the ‘index case’: but this, the search for identifying the first outbreak of a disease, is not the same as an index of endemicity – see (Steere-Williams, 2020).

9 These ideas were extensively reported in the *British Medical Journal* (Anon., 1901). Christophers repeated his assertion that eliminating malaria among the African population was an impossibility – preventing malaria among Europeans was, however, possible and practicable, particularly through deliberate segregation (Christophers, 1901). For more on segregation in general see (Cole, 2015; Curtin, 1985; Frenkel & Western, 1988; Nightingale, 2012; Spitzer, 1968).

10 There is a very convenient map of the incidence of malaria in this area around 1903 in (Sergent, 1903, page opposite titlepage). Another one, at a slightly later date, can be found in (Dedet, 2008, p. 223).

11 For more on Trolard, see (Loukas, Shea, Shea, Lutter-Hoppenheim, Zand, Tubbs & Cohen-Gadol, 2010). See also (Moulin, 1995; Strachan, 2006).

12 Called at the time anophelism without malaria, it concerned researchers for several decades. See (Fantini, 1994; Hackett, 1937, pp. 25–84).

13 Sergent was in favour of dispensing quinine as an added measure. It would be necessary, he argued, in a larger campaign (Sergent, 1903, p. 67).

14 Edmond Sergent knew and had read the reports of Koch’s observations (Sergent, 1903, pp. 88–89). Stephens and Christophers were also indebted to Koch, having already employed his endemic index (or endemicity) (Stephens & Christophers, (1902).

15 The brothers used the word “virus” to refer to the plasmodium rather than other words that were more familiar in the literature, such as parasite and haematozoa – even though they did use the word haematozoa when talking about the organism itself. David Bruce also used the words “reservoir” and “virus” in his study on sleeping sickness in cattle – see (Bruce, Hamerton, Bateman & Mackie, 1910). Whatever the reason they had for choosing “virus” they did not mean that the organism was a virus in the sense in which it is now understood in biology. See note 2, above.

16 The idea of quinine disinfecting the blood of the malarial subject was first expressed in (Sergent, 1903, p. 5) and kept popping up in their publications – for example (Sergent & Sergent, 1928, p. 132). The brothers recommended prophylactic administration of quinine to infected children. They realized that the delibrate separation of European and African was difficult: they recommended as little mixing as possible and the use of screens to protect the white population from mosquitoes. For more on the Sergent brothers’ anti-malarial methods over the decades, which is beyond the scope of this article, see (Dedet, 2008; Fredj, 2016; Sergent & Sergent, 1928; Sergent & Sergent, 1947).

17 See, for example, (Ross, 1902).

18 Lynteris (2022b) has recently shown that Simond’s experiments were “botched” and that he silenced about other doctors who defended the flea hypothesis before him, namely the Japanese Masanori Ogata.

19 This was the second British commission sent to India to study plague. On the first commission, see (Indian Plague Commission (1898–1899), 1901). On a comparison between both commissions, see (Evans, 2018).

20 Despite these apparent connections between malaria and plague, Tanon did not mention malaria at all in his works on plague. Indeed, Tanon referred to goats as examples of a disease reservoir, in that case of Malta fever (known today as brucellosis), an infectious disease he had previously studied (Tanon, 1922, p. 254, 1928, pp. 19–27). He also mentioned, in passing, rats as reservoirs of spirochaetosis (Tanon, 1922, p. 250). But this omission did not mean he ignored the work of the Sergent brothers. Given that Tanon taught about malaria at the Institute of Colonial Medicine, it is likely that he knew the research on malarial reservoirs (Tanon, 1928, p. 5).

21 We want to thank Christos Lynteris for sharing this source with us.
